# The R.M.O. and Emergency Calls from outside

**Published:** 1908-12-12

**Authors:** 


### Resident Medical Officers' Department.

### THE R.M.O. AND EMERGENCY CALLS FROM OUTSIDE.

[A correspondent sends us the following communication, which raises a question of considerable importance to resident medical officers. The writer deals very briefly with the whole subject, and leaves several interesting points in a condition ripe for further discussion. Moreover, the problem has other and wider aspects, not touched upon in the present article, which will probably occur at once to the minds of those who have been placed in similar circumstances. We propose to refer to this matter again in a future issue; and in the meantime we shall be pleased to consider the views of other correspondents, and to publish those that are suitable.—Editor.]

"A man has hanged himself in —— Street. Will you go?" Such a message was delivered to the writer (he being the nearest medical man) whilst engaged "screening" a child in order to localise a swallowed farthing. The child had dyspnœa. The house in which the hanging had occurred was only a stone's-throw distant from the hospital. Clearly one emergency could not be left for another—however urgent, interesting, and lucrative—outside. But had the call come at a disengaged moment how would the case have been altered? Some hospital committees in their rules for the resident medical officer state definitely that he shall not attend a call from outside; that he must devote himself solely to the duties of the institution. But other hospitals make no such rule for the resident. All hospitals, no doubt, have an unwritten rule to this effect, but is this binding in cases of the kind instanced above? Would the jury at the inquest on such a case acquit the R.M.O. if it came out in evidence that he had been called and would not go? How would the public regard him, and the institution of which he was head when it read in the press that he had refused to attend, and that by going he might possibly have saved life? This is one of many knotty questions requiring immediate decision and prompt action which occur in the experience of the resident medical officer who is single handed. Should there be a written rule, he must obey it and refuse to go. In the other case he needs the support and sympathy of the committee for which he works in whatever action he may take, supposing there has been no precedent to guide him. A middle course would be to go and take charge of the case until the police surgeon or other private practitioner arrived on the scene, and then hand over the case to him. This would prevent its being said that he took a fee out of the G.P.'s pocket; it would give a jury no cause for adverse comment, and the press no chance of sensationalism.

